# From fossils to future – Jane Francis on polar exploration and changing climates

**DOI:** 10.1242/bio.061973

**Published:** 2025-03-24

**Authors:** Katie Pickup

**Affiliations:** Reviews Editor at The Company of Biologists

## Abstract

Professor Dame Jane Francis is a palaeoclimatologist whose research has focused on studying fossil plants, particularly from polar regions, to understand past biodiversity and climate. She is the Director of the British Antarctic Survey (BAS) and Chancellor of the University of Leeds, UK. She was awarded the Polar Medal for her contributions to British polar research in 2002, only the fourth woman to receive this recognition at that time. Throughout her career, Jane has conducted numerous Antarctic and Arctic expeditions, camping in remote and extreme environments to collect data about the polar regions from the days of the dinosaurs when Antarctica and the Arctic were warmer and covered in forests. We met at the BAS headquarters in Cambridge, UK, sitting alongside her collection of petrified fossil wood. Here, we discuss what these fossil plants can tell us about climate, the importance of Antarctic research in understanding our changing world, and the benefits of open science in promoting collaboration and even reducing carbon emissions.


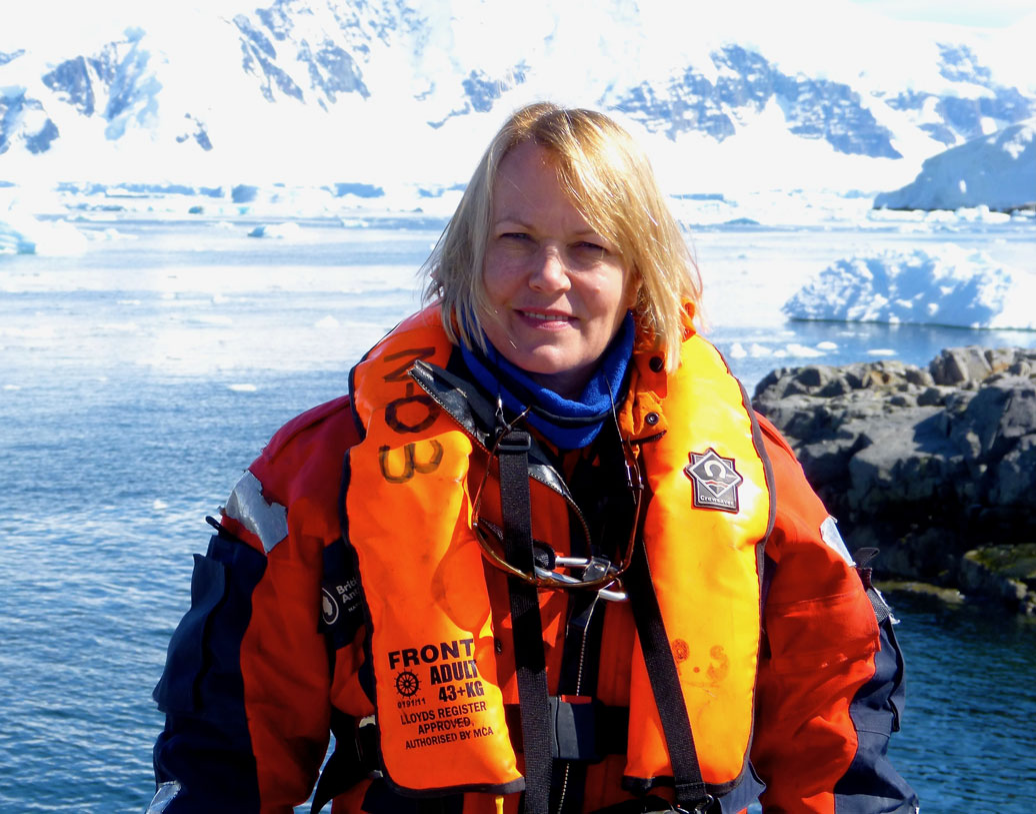


**Jane Francis**

## How much detail can be preserved in fossilised plant material?

I've collected fossil plants from the Jurassic Coast in Dorset, UK, and also from the Arctic and Antarctic. Many fossil leaves are mostly an impression of the leaf with just a thin veneer of carbonaceous material, but sometimes you can see great detail. You can see all the venation and features along the leaf margins in a well-preserved leaf – they look similar to modern-day leaves. In some fossil leaves, you can find cuticles with stomata preserved and different cell structures within the leaf are still visible.

Fossil wood is amazing because it's often petrified, which preserves the wood structure so well. When the wood was buried in sediment millions of years ago, it acted like a sponge to soak up mineral solutions that then crystallised within it, which often preserved all the delicate structural features of the wood. These include all the wall layers of the wood cells and all the information about the pitting that links one cell to another. If the wood is well preserved, I can make microscopic thin sections after cutting it with a diamond-tipped saw, and I can see the wood structure as you would see it in living wood.

## What can these details tell us about the climates these plants existed in?

Fossil plants, for me, are a tool to understand past environments. Palaeobotany – the study of fossil plants – is an important way by which we can understand what climate was like in the past. My research groups have looked at fossils of wood, leaves, flowers and pollen. Fossil pollen and spores can help date the rocks and are used for reconstructing past climates, along with the analysis of the geochemistry of the rocks and the types of sediments in which the fossils are preserved. All of these together can tell us about the environment and climate in the past. Comparison of the composition of the vegetation in the fossil record with that of similar modern vegetation today also helps us understand past climates. We know the climate tolerance of, for example, modern monkey puzzle trees or other Southern Hemisphere conifers, so we can use this information to guide reconstruction of past climates from similar fossil plants.

Other ways that palaeobotanists have developed our understanding of past climates is from features such as the leaf margins of fossilised flowering plants – whether they're toothed or smooth – which tells us a little about whether the climate was cold or warm. From tree ring patterns, we can determine growth rates and, for example, whether frost affected growth. Fossil tree rings can also show when there were periods of drought and when water shortage was a problem. In most Antarctic fossil wood, we can see that the trees grew well in warm, wet environments millions of years ago.

## What were these Antarctic forests like in the days before the continent was covered in ice?

The fossil plants that I've worked on were living around 70-100 million years ago, in a geological period called the Cretaceous when dinosaurs lived in these forests. Continents have moved around in the past due to tectonic activity, but from about 100 million years ago, the Antarctic continent moved over the South Pole and it's still in the same location today. So, if we see change in the climate based on the rock and fossil records, it is due to climate change. It's not due to continental movements.

The fact that we have fossil wood and leaves and other remains of plants and animals in the rocks in Antarctica when it was over the South Pole tells us that the climate was clearly much warmer. The climate at that point would have been warmed by natural carbon dioxide from volcanoes – so that was natural climate change. Antarctica was green. It was covered in many places with forests similar to modern Southern Hemisphere vegetation; these Antarctic forests were likely their ancestors. From the leaves, pollen and the fossil wood, we can identify monkey puzzle trees, which are very common in the fossil record from 100 million years ago, and other conifers. About 70 million years ago, angiosperms – flowering trees – became part of the vegetation.

Another interesting thing is that the plant fossils from polar latitudes that I've studied grew in a strange light environment. During field work in Antarctica today, we camp during summer months when there's no night – it's daylight for 24 hours. Then, in midwinter, it's dark all day. So, even if it was warm all year round in the past, these plants still had to cope with the unusual winter darkness and summer sunlight. And the plants couldn't migrate like dinosaurs probably did! Experiments that I've undertaken with colleagues in Australia on comparable modern plants suggest that during the Antarctic winter, it would have been cool and dark, and the plants probably became dormant to survive.

## Why is it so important to conduct research in Antarctica in the context of our modern-day climate?

Both polar regions are now responding to man-made warming and are the fastest changing environments on the planet. The Arctic is warming rapidly but Antarctica still has a much harsher, colder environment. When I gave talks 3 or 4 years ago, I said, “Antarctica is a big block of ice on a big block of rock, and it's not going to change very much”. We used the word ‘inertia’ a lot, thinking that Antarctica was going to take a long time to respond to climate change. But, in the last 2 years, we've seen considerable change in Antarctica. The important thing about studying Antarctica and the Arctic is that, although they're remote places at the ends of the Earth, what is happening there has a global impact. The ice is melting, and sea level is rising and threatening coastal regions across the planet. The oceans are also changing: the water that comes off Antarctica currently is dense and salty and drives the ocean circulation across the whole planet, linking it with the Arctic Ocean. As more freshwater from melting ice in Antarctica reaches the global ocean, the flow may change in the future, affecting the global climate.

## What changes have you personally experienced in the polar regions since you first started going on expeditions in the 1980s?

There are many places I've returned to that are now snow free, which reveal new rocks and fossils, but it's also worrying. There are islands around Antarctica where, in the past, people used to travel safely by skidoo from one island to another across the sea ice because it was frozen all year. By the time I went to Antarctica in the 1980s and 1990s, ice in the seaways between the islands had melted and ships could sail around islands, which they couldn't do previously.

Change in Antarctic sea ice is a major topic of concern at the moment. The ocean freezes around Antarctica and practically doubles the size of the continent every winter. But what we've seen in the last 2 years is a decrease in the sea ice each winter. This will have a profound effect on the ocean and global climate because the winter sea ice forms a white blanket that reflects the sun's heat and keeps the ocean cool. It's also home to many creatures that live there, such as krill, and algae. If the sea ice goes, the whole ecosystem structure will change.

**Figure:**
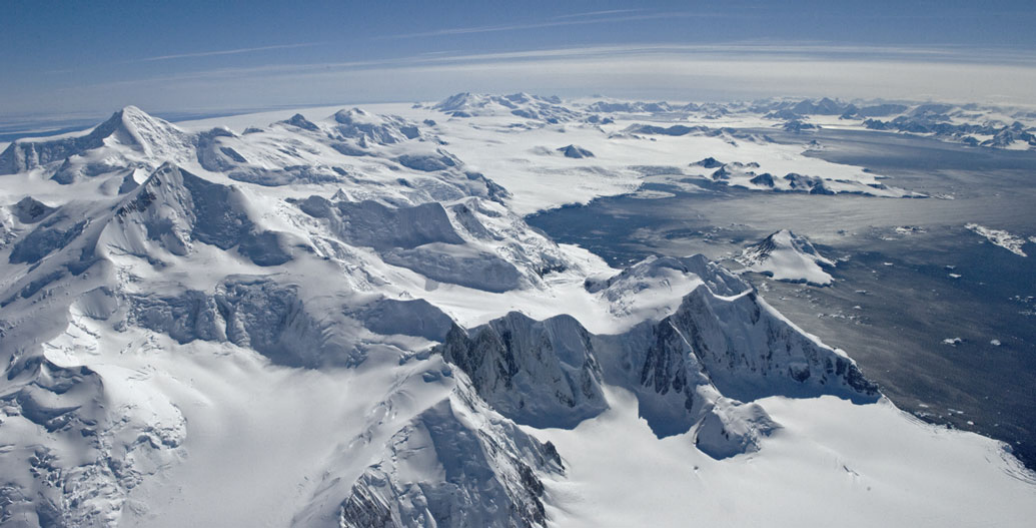
**Mountains in the Antarctic Peninsula.** Photo credits: British Antarctic Survey.

## Polar expeditions, particularly to Antarctica, have been very male dominated up until quite recently. What was your experience like as a woman in these environments?

I first went to Antarctica with scientists from New Zealand and the USA. I'd worked on fossil wood from Dorset, UK, and not many people had done that before – using fossil plants to understand climate from a geological context – so I was asked to work on fossil wood that had been found by early polar explorers, but no one had really understood its significance for palaeoclimate studies. The USA and New Zealand had women working in Antarctica then, but when I first worked for BAS in the 1980s, they didn't send women to camp in Antarctica. Women scientists stayed on ships, not in tents. It was only in the early 1990s that women from BAS were allowed to camp. There was a change in attitude to welcome a more diverse workforce and include more women, so it was a very positive move. Now there are far more women working in field camps in Antarctica.

## How did it feel to then become the first female Director of BAS in 2013?

I had worked in both the Arctic and the Antarctic, so I knew the UK and international Antarctic communities very well. Before I came to BAS, I was at the University of Leeds, UK, and I undertook field work in Antarctica for several months of the year. I knew BAS well because they had supported my field work, so it was a natural move for me. But, yes, being the first female Director was quite interesting. When I was unpacking my boxes in my new office during my first week, a helper asked if I was the new Director's secretary! Many times in the early days, people asked me who my boss was or which section of BAS I was directing. They couldn't quite believe that the Director was female.

Antarctica is not administered by a government; it's managed by members of the 58 countries that now have a presence in Antarctica. We have a meeting every year with all those nations to discuss how we work there to protect the environment and keep Antarctica for peace and science. I chaired one of the working groups – I was the first woman to do so. That was quite a challenge, working with many nations, some of which were probably not quite ready to accept a woman in an authoritative position.

## As a journal, Biology Open aims to support the principles of open science to promote transparency, sharing and inclusivity in research. How important are these principles to Antarctic and climate science?

Working in Antarctica is regulated by the Antarctic Treaty, which states that Antarctica is a continent for peace and science – so no mining, no warfare. One of the regulations is that all scientific results must be open and transparent. Everything that we publish is open and we have a polar data centre at BAS so that people can access the data. The Scientific Committee for Antarctic Research brings all the science together from nations with a presence in Antarctica to share and discuss our research. The whole structure of working in Antarctica is about openness and transparency. It's a huge continent to study with important science questions to investigate, which involve incredible logistical efforts, so it is important that the best science in Antarctica is done through international collaboration with results available to all.

## Is there anything publishers could do to better support the promotion of these open science principles?

Systems like having digital object identifiers (DOIs) for data have been helpful as it means data are published and can be traced more easily. Having the data published is important because it's so expensive to conduct research in Antarctica. We are also very concerned about our carbon footprint because Antarctic logistics include ships and aircraft that have a big impact; so, to reduce our carbon footprint, we aim to ensure that we work on the data that have been collected as fully as possible. Some grants now use data that have already been collected rather than requiring field work to collect new data. With better satellite transmission, we now have huge quantities of data about oceans, atmosphere, climate, ice sheets, etc. Having access to all the data is critical to help develop computer models that are used to understand how the Earth works, which are then used for forecasts and for policy.

## What's one key message that you think more people should be aware of in the context of climate change?

I think people must understand that we're now in a position where we have to adapt to our changing climate. The climate is changing fast now. The geological record tells us what the world was like in the past at different levels of carbon dioxide so that we can understand our future. It's important that we work in places like Antarctica and the Arctic to forecast how the climate will change so that we can prepare for the future. It's about adaptation, not just mitigation. And we seriously need to stop putting carbon dioxide into the atmosphere, otherwise we're going to go back to a world where forests will grow in Antarctica again. We know from the geological record that sea levels were much higher then and there was probably about half the extent of land exposed on Earth than there is now; so, if we returned to this past warm climate state, many coastal regions and islands would be lost. We need to be prepared to adapt to our rapidly changing climate and not just bury our heads in the sand, but, unfortunately, world politics are distracting efforts to focus on climate change at the moment.

## You have held many interesting positions throughout your career. Do you have any advice that you would like to pass onto early-career researchers?

You've got to be passionate about your science to be able to push the frontiers. The best thing I did was to work in Australia and get a different view of science – I learnt about new research topics from a different science community. If you can, experience as many different environments and network as much as possible to broaden your outlook on your field of research.

**Figure:**
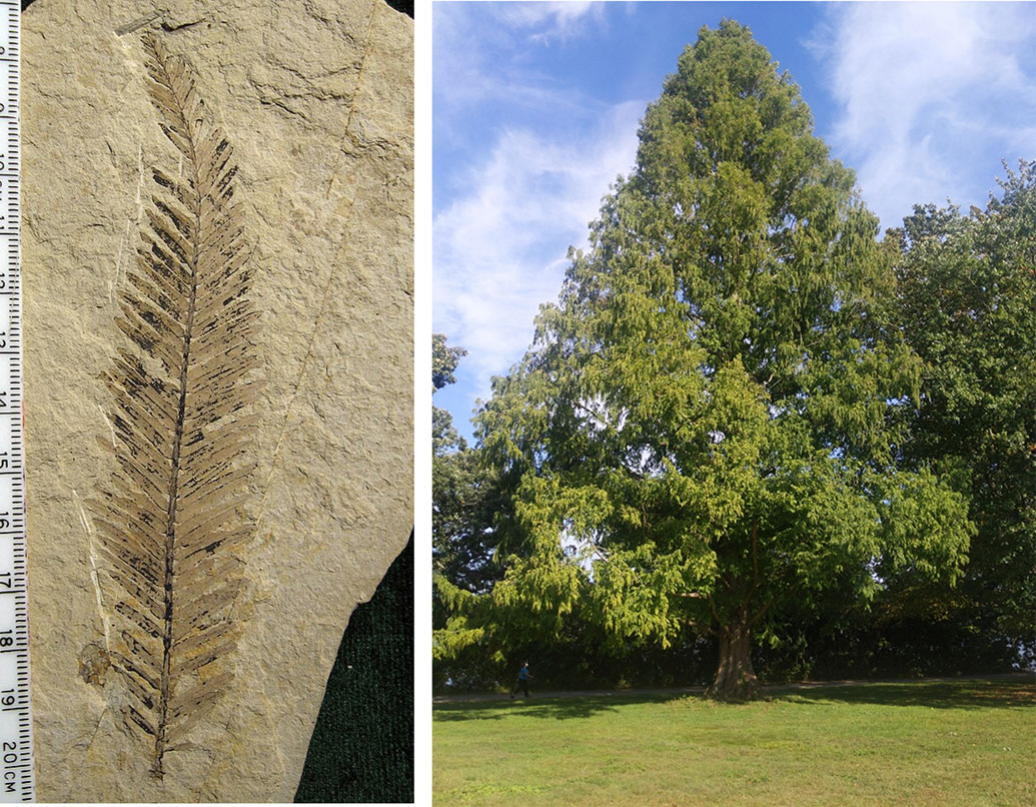
**A 49-million-year-old fossil leaflet from a *Metasequoia* (dawn redwood) tree (left) and a living *Metasequoia* tree (right).** Photo credits: Kevmin via Wikimedia Commons (left) and Peter A. Greenberg via iNaturalist (right) (published under a CC-BY 4.0 license).

## What do you like doing outside of your work?

I like gardening and I like growing plants that are living fossils, such as gingko and katsura, which are trees that used to grow in the Arctic millions of years ago. There are several trees that used to grow in past warm polar regions, which then disappeared when climates cooled and ice sheets formed. However, they survived at lower latitudes in special niches and are still growing today. About 45 million years ago, when the Arctic was warmer and covered in huge swamp forests, dawn redwoods (*Metasequoia*) used to grow across the region. As the ice set in, they disappeared from the Arctic and explorers later discovered them as fossils in the rock record, so the trees were assumed to have become extinct. But, in the 1940s, living *Metasequoia* were found growing in isolated valleys in China. Since then, they have been grown worldwide.

